# Case report: phlegmonous gastritis after endoscopic mucosal resection for a *Helicobacter pylori*-naïve foveolar-type gastric adenoma with a raspberry-like appearance

**DOI:** 10.1007/s12328-026-02322-3

**Published:** 2026-04-01

**Authors:** Kenichi Kishimoto, Kotaro Shibagaki, Yusuke Takahashi, Satoshi Kotani, Mamiko Nagase, Asuka Araki, Norihisa Ishimura, Daisuke Niino, Shunji Ishihara

**Affiliations:** 1 Department of Gastroenterology, Shimane University Hospital, Izumo, Japan; 2 Department of Endoscopy, Shimane University Hospital, 89 − 1 Enya, Izumo, 93–8501 Japan; 3 Department of Pathology, Shimane University Hospital, Izumo, Japan

**Keywords:** Phlegmonous gastritis, Endoscopic mucosal resection, Foveolar-type gastric adenoma with raspberry-like appearance, *Helicobacter pylori*, Complication

## Abstract

A 66-year-old man underwent endoscopic mucosal resection (EMR) for a small foveolar-type gastric adenoma with a raspberry-like appearance in *Helicobacter pylori*-naïve gastric mucosa. The following day, he developed high fever and septic shock with hemodynamic instability without abdominal pain. Contrast-enhanced computed tomography revealed localized gastric wall thickening from the fundus to the body, consistent with phlegmonous gastritis (PG) after EMR complicated by septic shock with hemodynamic instability. He was successfully managed conservatively with meropenem. *Streptococcus parasanguinis* and *Streptococcus salivarius* were subsequently isolated from the gastric juice.

PG is a rare but potentially fatal bacterial infection of the gastric wall that progresses rapidly. High fever is the most consistent finding, whereas abdominal pain may be absent, delaying recognition. Delayed diagnosis often leads to systemic sepsis and contributes to its high mortality. The main route of infection is direct mucosal invasion, often after endoscopic procedures, and most cases develop within one day of the intervention, with surgery frequently required. When high fever develops within a few days after endoscopic intervention, PG should be considered in the differential diagnosis, even in the absence of abdominal pain, to ensure timely management.

## Introduction

Phlegmonous gastritis (PG) is a rapidly progressive, suppurative infection of the stomach characterized by bacterial invasion of the submucosa with extension into all layers of the gastric wall. PG is a rare disease but is often fatal if not promptly diagnosed and treated. Reported routes of infection include direct invasion through injured gastric mucosa, hematogenous spread from distant sites, and lymphatic dissemination from adjacent septic foci [1]. Direct mucosal invasion is particularly noted after endoscopic procedures, most commonly endoscopic submucosal dissection for early gastric cancer.

The recent decrease in *Helicobacter pylori* (*Hp*) prevalence has led to a decline in gastric cancer overall, whereas reports of *Hp*-naïve gastric neoplasms (HpNGN) have been increasing. Among these, foveolar-type gastric adenoma with a raspberry-like appearance (FGA-RA) is a small polyp and represents the most common histologic subtype of HpNGNs [2], usually treated by endoscopic mucosal resection (EMR). Here, we describe a rapidly progressive case of severe PG that developed after EMR of a small FGA-RA.

## Case report

A 66-year-old man was admitted to our department for endoscopic resection of a gastric polyp. His medical history included diabetes mellitus and non-alcoholic fatty liver disease. His regular medication was dapagliflozin. He was not taking systemic corticosteroids, proton pump inhibitors, or other immunosuppressive agents, and he had no underlying immunocompromising conditions or evidence of malnutrition. He denied alcohol consumption but had a 36-year history of smoking 30 cigarettes per day. Physical examination was unremarkable. Laboratory tests on admission revealed mildly elevated liver enzymes and an HbA1c level of 6.6% (NGSP), with no other abnormalities. Serum *Hp*-IgG antibody and fecal *Hp* antigen were both negative, indicating an *Hp*-naïve status.

**Fig. 1:**
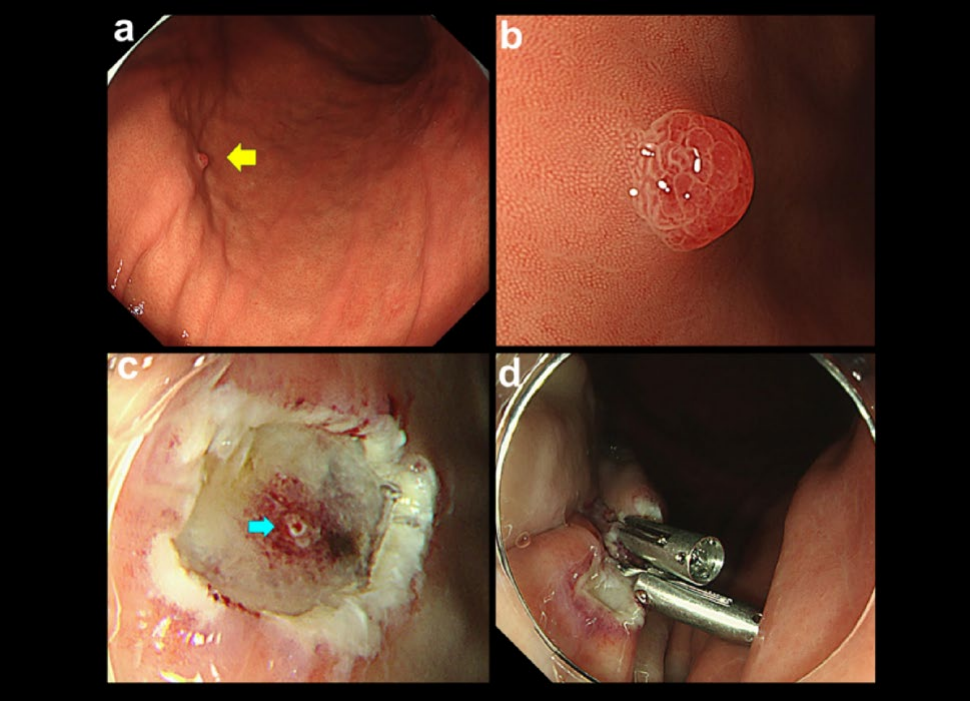
**a** Endoscopic view showing a small, reddish polyp (yellow arrow) on non-atrophic, *Helicobacter pylori*-naïve fundic gland mucosa. **b** Magnified observation revealed an irregular papillary surface, characteristic of a foveolar-type gastric adenoma with a raspberry-like appearance. **c** After cap-assisted endoscopic mucosal resection (EMR), an exposed vessel (blue arrow) was visible on the resection bed. **d** The resected ulcer was completely closed with clips to prevent delayed bleeding

Esophagogastroduodenoscopy revealed a 3 × 3 mm reddish polyp on the greater curvature of the mid-gastric body, on non-atrophic, *Hp*-naïve fundic gland mucosa (Fig. 1a). Magnified observation showed an irregularly shaped papillary structure with a clear demarcation from the surrounding mucosa (Fig. 1b), findings suggestive of an FGA-RA. After submucosal injection of saline containing diluted epinephrine (0.5 mg in 100 mL), cap-assisted EMR was performed using a crescent-shaped snare. Resection was performed using an electrosurgical unit (VIO 300D; ERBE; Elektromedizin, Tübingen, Germany) with SwiftCoag mode (30 W, effect 4), and the lesion was resected en bloc without intraprocedural bleeding. An exposed vessel was identified on the resection bed (Fig. 1c), and the mucosal defect was completely closed with endoscopic clips to prevent delayed bleeding (Fig. 1d).

**Fig. 2:**
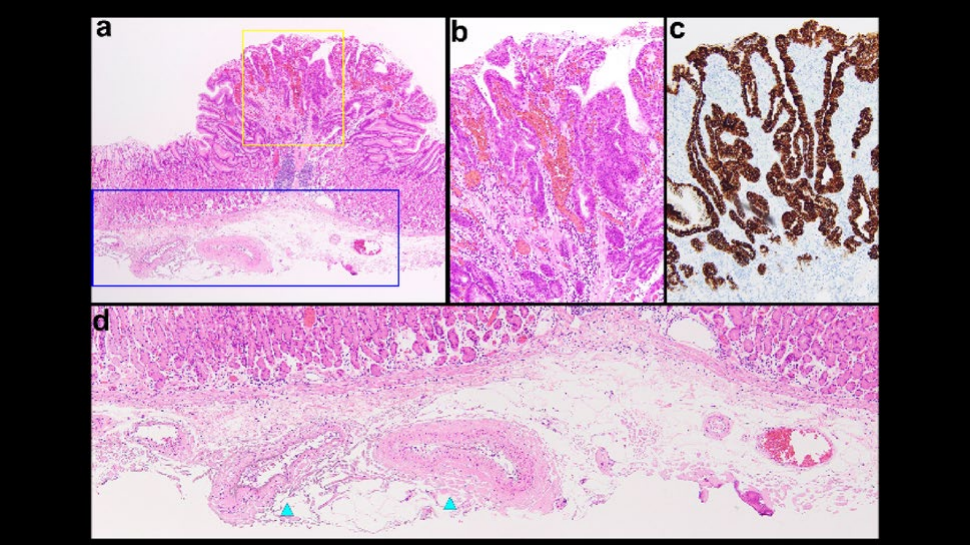
**a** Neoplastic epithelium proliferating upward on fundic gland mucosa. **b** Irregular papillary glands with stratified nuclei, loss of polarity, and indistinct mucin cap, consistent with high-grade dysplasia. **c** Strong, diffuse MUC5AC expression indicating foveolar differentiation, confirming foveolar-type adenoma with raspberry-like appearance. **d** The small vessels were identified in the submucosal layer (blue arrowheads)

Histologically, the lesion demonstrated upward neoplastic proliferation overlying the fundic gland mucosa (Fig. 2a). It consisted of an irregular papillary glandular architecture with markedly stratified nuclei, loss of polarity, and an indistinct mucin cap (Fig. 2b). Strong, diffuse MUC5AC expression was observed (Fig. 2c), consistent with a foveolar-type adenoma exhibiting high-grade dysplasia. A small submucosal vessels (blue arrowheads) corresponded to that identified endoscopically (Fig. 2d).

**Fig. 3:**
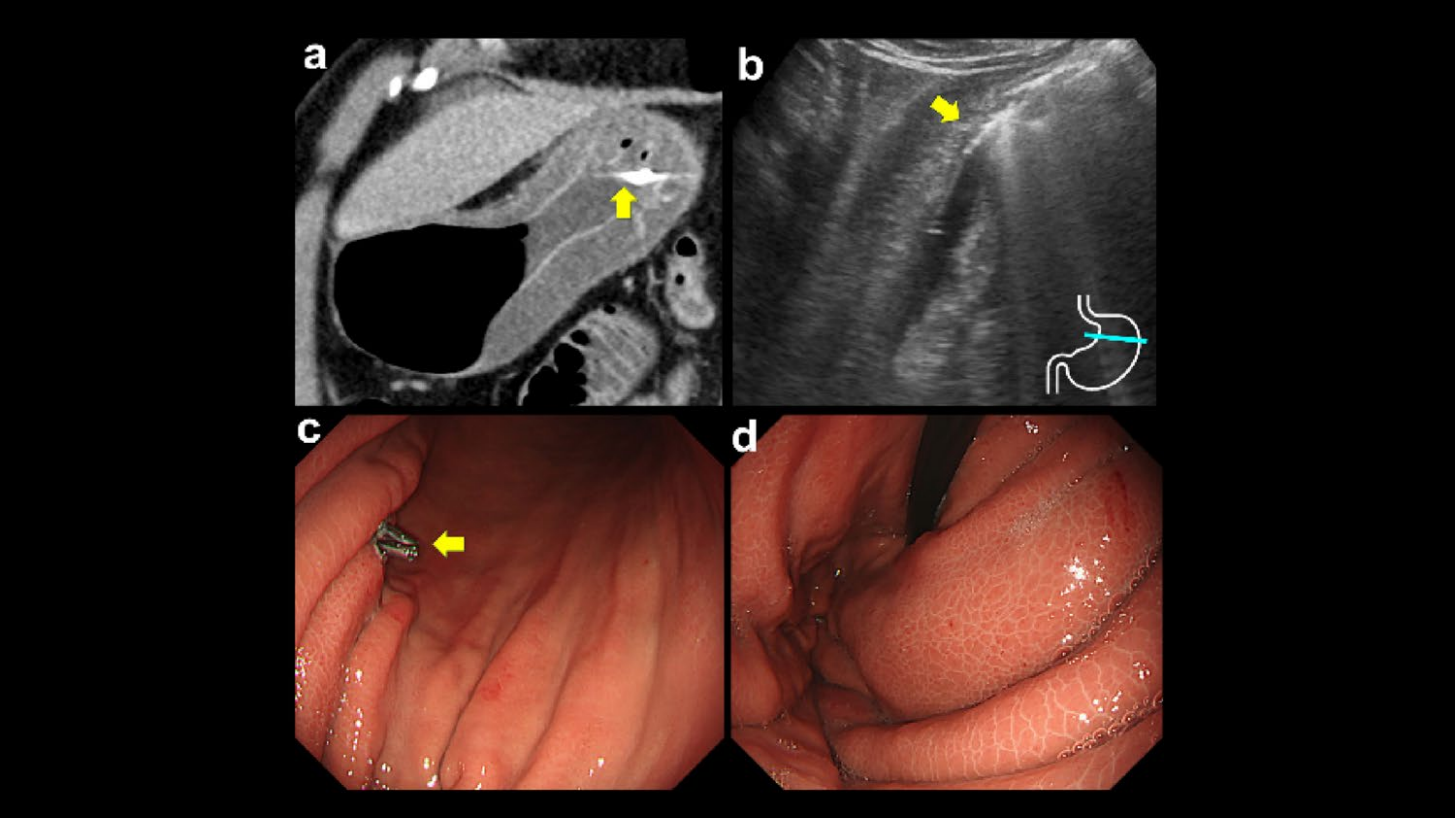
**a** Contrast-enhanced computed tomography (CT) the day after endoscopic mucosal resection (EMR) showed segmental circumferential gastric wall thickening from the fundus to the body at the clipped site (yellow arrow), suggesting phlegmonous gastritis (PG). **b** Ultrasonography confirmed circumferential thickening with preserved layers and marked submucosal edema (the yellow arrow indicates the clip placed during EMR). **c** Endoscopy on day 6 showed gastric body wall thickening near the resection site closed by clipping (yellow arrow). **d** Similar mucosal changes in the fornix indicated healing phase of PG

**Fig. 4:**
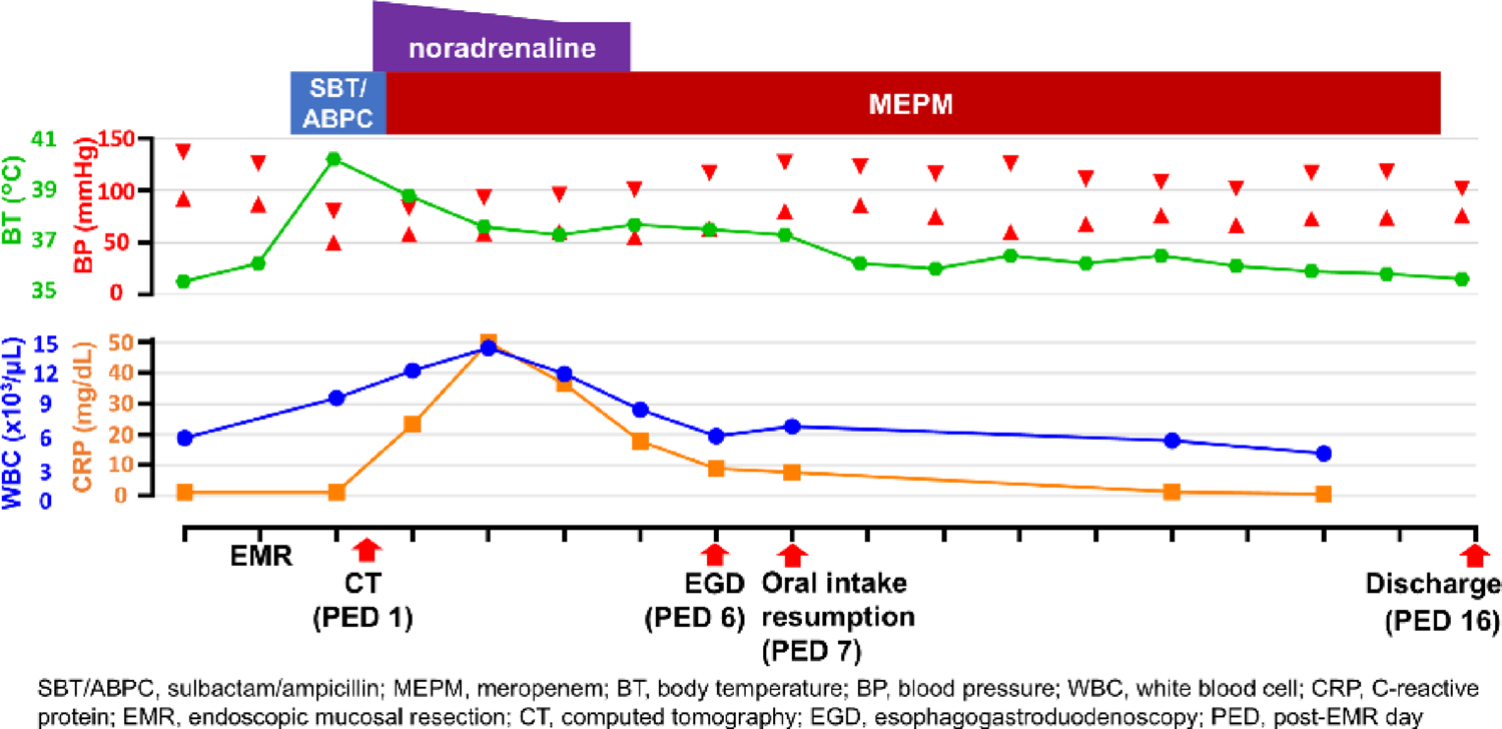
Clinical course of the patient

On the day after EMR, the patient developed a high fever without abdominal pain, and aspiration pneumonia was initially suspected; therefore, sulbactam/ampicillin therapy was initiated at a dose of 3 g every 6 h. However, his systolic blood pressure abruptly dropped below 80 mmHg accompanied by tachycardia and diaphoresis, indicating septic shock with hemodynamic instability. Laboratory tests showed leukocytosis, elevated CRP and procalcitonin levels, and acute kidney injury, consistent with septic shock (Table 1). Although acute kidney injury was present, priority was given to evaluating the focus of infection. After adequate intravenous hydration, contrast-enhanced abdominal computed tomography (CT) was performed and demonstrated segmental circumferential gastric wall thickening from the fundus to the body, centered on the clipped resection site (Fig. 3a). Ultrasonography (US) showed corresponding circumferential wall thickening in the same region, with preserved wall stratification and marked submucosal edema (Fig. 3b). Based on these findings, PG with septic shock was diagnosed. Gastric juice culture and two sets of blood cultures were obtained, and the antibiotic was switched to meropenem at a dose of 1 g every 8 h for broader coverage. *Streptococcus parasanguinis* and *Streptococcus salivarius* were subsequently isolated from the gastric juice and were intermediate to sulbactam/ampicillin and susceptible to meropenem, so antibiotic therapy was continued. Both sets of blood cultures were negative. The patient’s general condition improved with antibiotics and intravenous hydration. Follow-up endoscopy on post-EMR day 6 revealed circumferential gastric wall thickening from the fundus to the body, consistent with healing phase PG (Fig. 3c, d). Oral intake was resumed the next day, and the patient remained free of abdominal pain throughout the clinical course; therefore, he was discharged on post-EMR day 16. Meropenem was administered for a total duration of 14 days, and no oral step-down therapy was performed. The clinical course is shown in Fig. 4.

**Table 1:** Laboratory data

WBC	11.9 × 10^9^	/L		AST	20	U/L
Neutrophils	91	%		ALT	27	U/L
Monocytes	2.5	%		LDH	172	U/L
Lymphocytes	1.5	%		ALP	50	U/L
RBC	4.38 × 10^12^	/L		GGT	38	U/L
Hemoglobin	14	g/dL		Amylase	45	U/L
Hematocrit	41.6	%		BUN	32	mg/dL
Platelet	127 × 10^9^	/L		Creatinine	2.09	mg/dL
PT	67.6	%		plasma glucose	202	mg/dL
PT-INR	1.23			Sodium	135	mEq/L
APTT	31	s		Potassium	4.5	mEq/L
D-dimer	4.6	µg/mL		Chloride	105	mEq/L
Total protein	5.1	g/dL		Calcium	7.3	mg/dL
Albumin	2.7	g/dL		CRP	23.19	mg/dL
Total bilirubin	1.1	mg/dL		procalcitonin	53	ng/mL

WBC, white blood cells; RBC, red blood cells; PT, prothrombin time; PT-INR, PT-international normarized ratio; APTT, activated partial thromboplastin time; AST, aspartate aminotransferase; ALT, alanine aminotransferase; LDH, lactate dehydrogenase; ALP, alkaline phosphatase; GGT, γ-glutamyl transpeptidase; BUN, blood urea nitrogen; CRP, C-reactive protein0

## Discussion

**Fig. 5:**
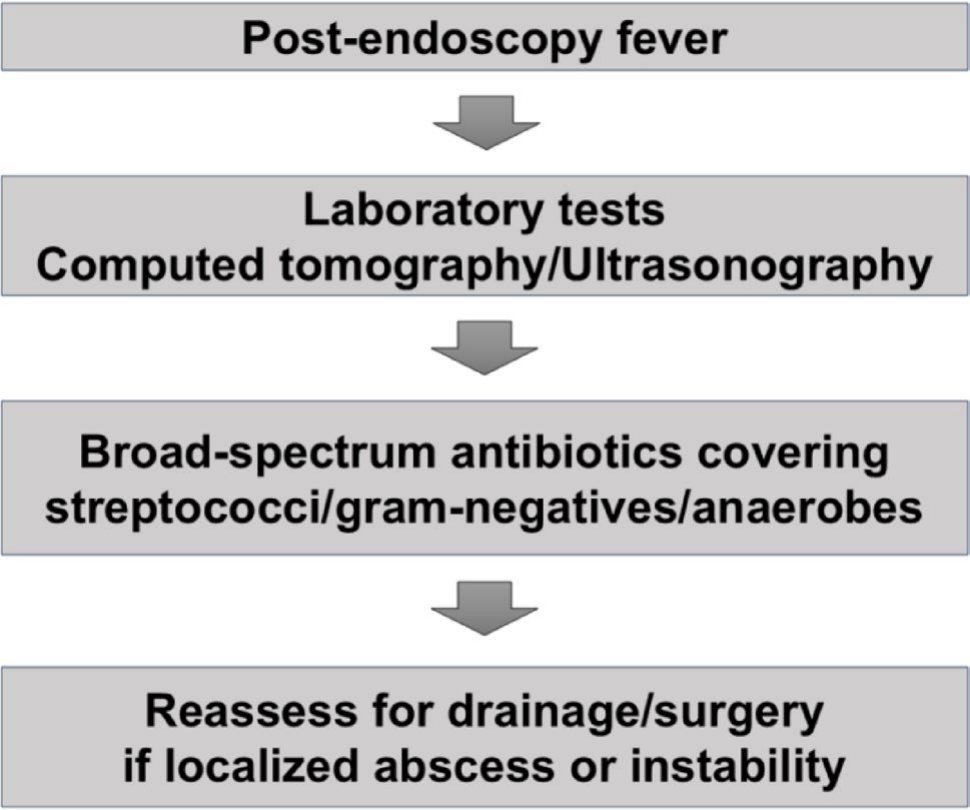
Early recognition and management of phlegmonous gastritis after endoscopic procedure

PG is a rare but potentially fatal bacterial infection of the gastric wall that progresses rapidly. It is classified as diffuse or localized type, with the diffuse type having a higher mortality rate than the localized form [3]. Delayed diagnosis often leads to systemic sepsis, which contributes to its high mortality (27–42%) [3, 4]. Clinically, high fever is consistently present, although abdominal pain may sometimes be absent, potentially hindering early recognition [5]. In this case, the inflammation was confined to the EMR site, consistent with the localized type. Fever occurred without abdominal pain, delaying clinical suspicion of PG; diagnosis was made only after septic shock with hemodynamic instability developed, leading to further diagnostic work-up. Earlier CT evaluation at the time of the initial fever might have facilitated earlier diagnosis and potentially prevented clinical deterioration. Although several conditions may mimic PG after endoscopic procedure, the diagnosis in the present case was readily narrowed by the acute clinical course and CT/US findings (Table 2) [3, 6–13]. Because the onset occurred during hospitalization, antimicrobial therapy was promptly escalated thereafter, which may have contributed to successful conservative management. This case highlights that high fever after gastric endoscopic procedure should prompt consideration of PG even in the absence of abdominal pain. A practical clinical algorithm of high fever after endoscopic procedure is summarized in Fig. 5.

**Table 2:** Differential diagnosis of phlegmonous gastritis

Disease	CT findings	EUS/US findings	Clinical features	Recommended next tests / steps	Refs.
Phlegmonous gastritis	Gastric wall thickening, submucosal edema; centered on recent endoscopic resection site	Gastric wall thickening with preserved layer stratification	High fever; abdominal pain; can rapidly progress to sepsis/shock	Blood/gastric juice cultures; broad-spectrum antibiotics; consider drainage/surgery	[3]
Emphysematous gastritis	Gas within gastric wall, wall thickening; portal venous gas	Reverberation artifacts from intramural gas	severe sepsis; often severe abdominal pain; high mortality	Emergency surgical consult; broad-spectrum antibiotics	[6]
Benign gastric emphysema	Intramural gas but mild wall thickening, no portal venous gas	Intramural gas artifacts	Often mild symptoms; occurs after vomiting/trauma/endoscopy	Conservative management; exclude ischemia/emphysematous gastritis	[6]
Intramural abscess	Focal intramural low-density collection; localized wall thickening	Focal hypoechoic/anechoic collection within wall	Fever; localized pain	Consider endoscopic drainage if feasible; antibiotics	[7]
Linitis plastica	Diffuse wall thickening with poor distensibility; enlarged nodes	Layer disruption; loss of normal stratification	Chronic course (weight loss, anorexia)	Multiple biopsies; staging CT; tumor markers adjunct	[8]
Gastric lymphoma	Segmental/diffuse thickening; bulky folds; lymphadenopathy	Hypoechoic thickening; loss of stratification	unexplained fever, drenching night sweats, unintentional weight loss	Multiple biopsies; PET/CT; hematology consult	[9]
Ischemic gastritis	Poor mucosal enhancement; vascular disease findings	Variable; may show thickening with compromised perfusion	Severe pain; vascular risk; shock can be cause or consequence	Evaluate vasculature with computed tomography angiography	[10]
Corrosive gastritis	Wall thickening; mucosal hyperenhancement	Edema; may show deep injury with layer disruption	History of ingestion; oral/pharyngeal lesions possible	Supportive care; surgery if perforation/necrosis	[11]
Post-ESD/EMR coagulation syndrome	Mild localized wall thickening; changes adjacent to resection site	Superficial inflammatory change; preserved layers	Fever and localized pain/tenderness	Fasting, fluids, analgesia, antibiotics	[12]
Microperforation	Extraluminal/free air, focal fat stranding; possible localized fluid	May show focal wall defect or peritoneal irritation signs	Post-procedural pain, peritoneal signs; fever variable	Antibiotics; endoscopic closure or surgery depending on severity	[13]

CT, computed tomography; EUS, endoscopic ultrasonography; US, ultrasonography; PET/CT, positron emission tomography/computed tomography

Known risk factors for PG include immunodeficiency, gastric carcinoma, chronic alcohol use, achlorhydria, diabetes, malnutrition, and prolonged glucocorticoid therapy. Previous reports have also implicated endoscopic procedures as predisposing events [5]. In this case, diabetes was well controlled and no other immunocompromising conditions were present, suggesting that EMR itself was the principal predisposing event.

The causative organisms of PG are diverse, but *Streptococcus* species are most frequently implicated, accounting for 57–68% of reported cases [5, 14]. Among them, *S. pyogenes* is the most commonly isolated pathogen [1]. Although these are commensal oral streptococci, under the risk factors described above they can act as causative agents of PG. In our case, *S. parasanguinis* and *S. salivarius* were isolated from gastric juice, consistent with previous reports, although blood cultures were negative. Swallowed oral streptococci likely penetrated the gastric wall and submucosal vessels through the post-EMR ulcer, causing rapid intramural spread and presumed bacteremia, which led to septic shock with hemodynamic instability. Although prophylactic closure of the post-EMR defect may theoretically reduce bacterial translocation, evidence for its protective effect against infectious complications is limited. In our case, PG developed despite complete clip closure, suggesting that defect closure alone may not be sufficient to prevent this rare complication.

To date, 17 cases of PG following endoscopic procedures have been reported, identified through a literature search of PubMed and Ichushi (Medical Central Journals in Japan) (Table 3) [7, 15–28]. Symptom onset occurred within one day after the procedure in 59% (10/17) of cases, underscoring the typically rapid clinical course. Endoscopic submucosal dissection (ESD) was the most common precipitating procedure (53%, 9/17), followed by biopsy (24%, 4/17), EMR (12%, 2/17, including the present case), and other interventions such as endoscopic ultrasound-guided fine-needle aspiration and argon plasma coagulation. Surgical management was required in 65% (11/17) of patients, whereas 29% (5/17) were successfully treated with antibiotics alone; one additional localized case was managed by endoscopic drainage. Overall mortality was 12% (2/17). Notably, all reported cases of localized type PG (4/4) resulted in recovery with diverse management strategies (surgery, antibiotics, or endoscopic drainage), whereas diffuse type PG more frequently necessitated surgical intervention and accounted for all fatal outcomes, suggesting worse prognosis in the diffuse form.

**Table 3:** Reported cases of phlegmonous gastritis after endoscopic procedures

Case	Age	Sex	Hp-infection	Target lesion	Procedure	Symptom	type	Time to onset	Comorbidities	Treatment	Outcome	Refs.
1	68	M	N/A	gastric erosion	biopsy	fever, abdominal pain	diffuse	3 days	HTN, DM	gastrectomy	cure	[15]
2	68	F	N/A	gastric adenoma	EMR	fever, abdominal pain	diffuse	1 day	none	gastrectomy	cure	[16]
3	50s	M	N/A	early gastric cancer	ESD	fever, abdominal pain	diffuse	2 days	HTN	antibiotics	cure	[17]
4	74	M	N/A	early gastric cancer	ESD	fever, abdominal pain	diffuse	1 day	DM, chronic kidney disease	gastrectomy	cure	[18]
5	93	M	N/A	early gastric cancer	ESD	fever, abdominal pain	diffuse	1 day	none	gastrectomy	cure	[19]
6	70	F	N/A	pancreatic tumor	EUS-FNA	fever, abdominal pain	diffuse	7 days	pancreatic cancer	antibiotics	cure	[20]
7	62	M	N/A	advanced gastric cancer	biopsy	fever, abdominal pain	diffuse	4 days	HTN, gastric cancer	gastrectomy	death	[21]
8	58	M	N/A	early gastric cancer	ESD	fever, abdominal pain	diffuse	1 day	alcoholic liver disease	gastrectomy	cure	[22]
9	91	M	N/A	early gastric cancer	ESD	fever, abdominal pain	localized	1 day	none	gastrectomy	cure	[22]
10	56	F	N/A	gastric vascular ectasia	APC	fever, abdominal pain	diffuse	2 days	Sjögren’s syndrome	gastrectomy	death	[23]
11	63	F	N/A	early gastric cancer	ESD	Abdominal pain	localized	5 days	none	endoscopic drainage	cure	[7]
12	91	F	N/A	early gastric cancer	ESD	fever	diffuse	1 day	HTN	antibiotics	cure	[24]
13	76	M	N/A	early gastric cancer	ESD	fever	diffuse	1 day	DM, myelodysplastic syndromes	antibiotics	cure	[25]
14	67	M	N/A	normal mucosa	biopsy	fever, abdominal pain	diffuse	1 day	DM	gastrectomy	cure	[26]
15	72	F	positive	early gastric cancer	ESD	none	loclized	10 weeks	HTN	gastrectomy	cure	[27]
16	76	M	positive	gastritis	biopsy	fever, abdominal pain	diffuse	1 day	none	gastrectomy	cure	[28]
17	**66**	**M**	**naïve**	**gastric adenoma (FGA-RA)**	**EMR**	**fever**	**localized**	**1 day**	**DM**	**antibiotics**	**cure**	**our case**

M, male; F, female; Hp, Helicobacter pylori; N/A, not available; FGA-RA, foveolar-type gastric adenoma with a raspberry-like appearance; EMR, endoscopic mucosal resection; ESD, endoscopic submucosal dissection; EUS-FNA, endoscopic ultrasound guided fine needle aspiration; APC, argon plasma coagulation; HTN, hypertension; DM, diabetes mellitus. Our case is indicated in bold.

Given the low incidence of PG, the presence of risk factors alone should not preclude endoscopic treatment. Nevertheless, because FGA-RA, an *Hp*-naïve gastric neoplasm that histologically shows irregular papillary glandular architecture and a pure foveolar-type gastric phenotype with exclusive MUC5AC expression, is a non-invasive lesion with negligible lymph-node metastatic risk [29], even rare procedural risks such as PG should be considered when planning endoscopic treatment.

In conclusion, PG is a rare but potentially fatal condition that can occur as a complication of endoscopic procedures. When high fever develops after an endoscopic procedure, PG should always be considered in the differential diagnosis to ensure timely diagnosis and management.

## Abbreviations

CT: Computed tomography
EMR: Endoscopic mucosal resection
FGA-RA: Foveolar-type gastric adenoma with a raspberry-like appearance
*Hp*: *Helicobacter pylori*
HpNGN: *Hp*-naïve gastric neoplasms
PG: Phlegmonous gastritis
